# Decoding the Cauzin Softstrip: a case study in extracting information from old media

**DOI:** 10.1007/s10502-021-09358-z

**Published:** 2021-02-25

**Authors:** Michael Reimsbach, John Aycock

**Affiliations:** 1 Saarland, Germany; 2 Department of Computer Science, University of Calgary, 2500 University Drive N.W., Calgary, AB T2N 1N4 Canada

**Keywords:** Cauzin Softstrip, Barcode, Optical recognition, Convolutional neural network, Deep learning

## Abstract

Having content in an archive is of limited value if it cannot be read and used. As a case study of extricating information from obsolete media, making it readable once again through deep learning techniques, we examine the Cauzin Softstrip: one of the first two-dimensional bar codes, released in 1985 by Cauzin Systems, which could be used for encoding all manner of digital data. Softstrips occupy a curious middle ground, as they were both physical and digital. The bar codes were printed on paper, and in that sense are no different in an archival way than any printed material. Softstrips can be found in old computer magazines, computer books, and booklets of software Cauzin produced. However, managing the digital nature of these physical artifacts falls within the scope of digital curation. To make the information on them readable and useful, the digital information needs to be extracted, which originally would have occurred using a physical Cauzin Softstrip reader. Obtaining a working Softstrip reader is already extremely difficult and will most likely be impossible in the coming years. In order to extract the encoded data, we created a digital Softstrip reader, making Softstrip data accessible without needing a physical reader. Our decoding strategy is able to decode over 91% of the 1229 Softstrips in our Softstrip corpus; this rises to 99% if we only consider Softstrip images produced under controlled conditions. Furthermore, we later acquired another set of 117 Softstrips and we were able to decode nearly 95% of them with no adjustments to the decoder. These excellent results underscore the fact that technology like deep learning is readily accessible to non-experts; we obtained these results using a convolutional neural network, even though neither of the authors are expert in the area.

## Introduction

Home computers in the late 1970s and early 1980s were becoming more affordable and targeting the mass market instead of computer enthusiasts, but still required a lot of technological knowledge. For instance, computer owners’ manuals typically included information about BASIC programming, teaching users how to program their own software (e.g., Apple Computer 1979). It was also common for computer magazines to contain software in the form of source code—type-in programs. In order to use the programs, people had to manually enter the code, and even a single mistake could result in an error.

To make matters worse, storage devices were usually not part of a computer and had to be purchased separately. Cassette tape decks were one option, albeit slow, and floppy disk drives were available but costly. A potential solution came in the form of bar codes. Two of the most widely recognized bar codes today are the Universal Product Code (UPC) for identifying products and the Quick Response (QR) code which is used in many different areas such as manufacturing, health care, and marketing (Denso 2012).

**Fig. 1:**
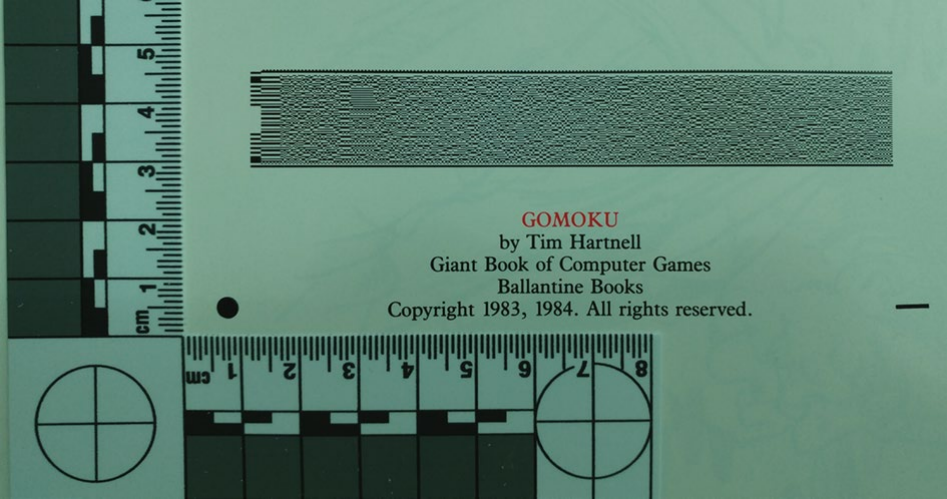
An example Softstrip

By contrast, the Cauzin Softstrip shown in Fig. 1 is an almost-forgotten relic, a two-dimensional bar code format released in 1985 by Cauzin Systems (Sandberg-Diment 1985). All kinds of digital data, such as graphics, software or text files, could be encoded as a Cauzin Softstrip and then printed on paper.

Cauzin’s optical reader, the Softstrip System Reader, sold for approximately 200 USD (Baskin 1987; Johnson 1986), with Cauzin’s encoding software—dubbed the “Stripper”—retailing at under 30 USD (Baskin 1987; Cauzin Systems, Inc (n.d.a)). It was compatible with the IBM PC, Apple II, and Macintosh, and in fact Softstrips could be used to transfer data between these different platforms (Cauzin Systems, Inc (n.d.b)). Softstrips appeared in magazines, were sold in stores, and appeared in at least one book. It provided an inexpensive software distribution medium for publishers.

Although MacUser magazine proclaimed the Cauzin Softstrip the most innovative concept of 1986 (MacUser 1987) and Cauzin Systems had plans to use the Cauzin Softstrip on cards such as credit or calling cards (Glaberson and Santulli 1989), the technology was not as successful as anticipated and eventually disappeared a few years after its release in 1985. Floppy disks became cheaper and more widespread, and programs became larger; the Softstrip is impractical for larger data, with a single Softstrip only able to store 5500 bytes (Cauzin Systems, Inc 1986a; Cauzin Systems, Inc (n.d.a); Johnson 1986). By contrast, an Apple II floppy disk from that time would hold over 25 times as much data. Larger data items could be split across multiple Softstrips, but this increased scanning time (already 30 s for a full strip) and required the user to adjust the Softstrip Reader again for each Softstrip (Johnson 1986).

It is already difficult to find a Cauzin Softstrip Reader, working or otherwise. To underscore this point, we were able to acquire one on eBay only *after* this work was complete, and even then the software is missing. Further, the mechanical nature of a Cauzin reader suggests that long-term functionality is not guaranteed. The only other option to decode the Softstrips without access to a Cauzin Softstrip Reader is to do it by hand, a time-consuming and error-prone task. By way of illustration, a Softstrip had to be decoded manually for this work to locate a decoding error, a process that took about eight hours until the decoding was successful. Use of a Softstrip Reader, of course, is predicated on the assumption that an appropriate “accessory kit” is found to interface the Reader to one of the supported host computers (for instance, either a serial port or a cassette port was used to connect to the Apple II, depending on the computer model), that a decades-old host computer is available and functioning, and that a mechanism for exporting data off the host computer exists.

This is the situation we found ourselves in, one doubtless familiar to memory institutions and software preservationists, where we had data locked in an obsolete format—here, we had physical Softstrips with data on them but no physical reader. How could we access this data and allow it to be assessed, researched, and ultimately preserved? Instead of compounding our obsolescence problems by trying to find a physical reader and cajole it back into life, we applied modern technology. We created a digital optical reader using deep learning, demonstrating in the process that deep learning is a technique within reach of non-experts.

## Anatomy of a Softstrip

This section explains the structure of the Cauzin Softstrip and how digital data is encoded within it. The Cauzin Systems patents (Brass et al. 1987, 1988a, b) were particularly helpful for understanding the details of the Softstrip format, and the information here is drawn from them unless stated otherwise.

**Fig. 2:**
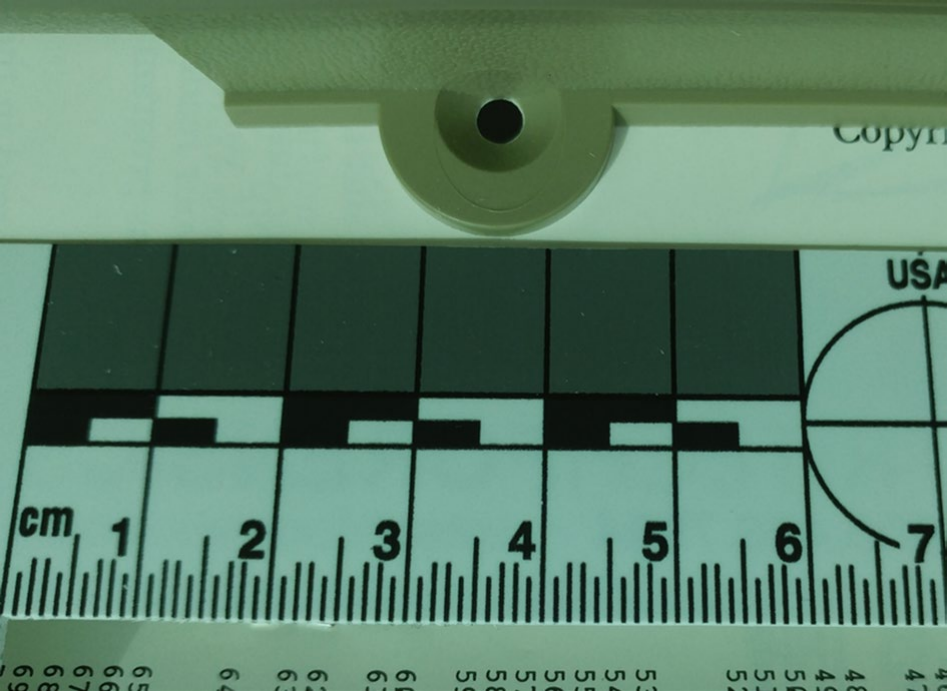
Softstrip aligned in the reader

### Basics

A Softstrip is 5/8 inches wide and up to 10 inches long (Brass et al. 1987; Johnson 1986), with two positioning marks for the Softstrip reader: a circle in the upper left and a rectangle in the lower left (Fig. 1). Figure 2 shows how the example strip looks when aligned in the reader.

**Fig. 3:**
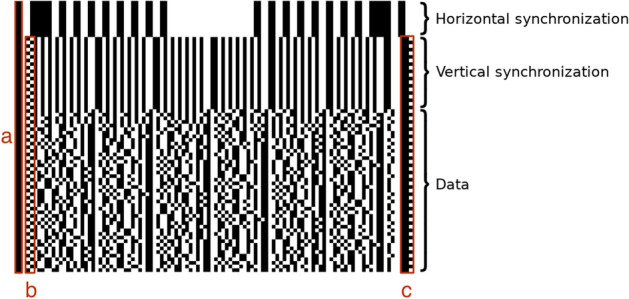
Softstrip structure, with: **a** start bar; **b** checkerboard; **c** rack

Each Softstrip is divided into three sections (Fig. 3). The first part is the horizontal synchronization section, the second is the vertical synchronization section and the last part contains the strip’s data. The two synchronization sections are collectively referred to as the *header* and contain encoded metadata about the Softstrip itself.

**Fig. 4:**
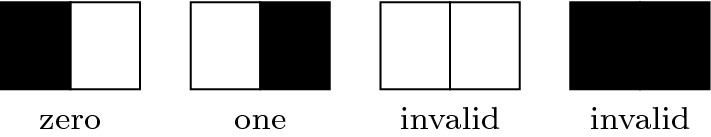
Possible dibit values

The Cauzin Softstrip uses two adjacent squares, called a *dibit*, for encoding a single data bit. A zero data bit is encoded by a black square followed by a white square, whereas a one data bit is encoded by a white square followed by a black square; other combinations are invalid (Fig. 4).

The start of the Softstrip is indicated by a one-dibit-wide black bar on the left side (Fig. 3a), followed by one white square. Both the checkerboard and rack (Fig. 3b, c respectively) change each row and are used to determine the start and end of a row.

Data are located in between the checkerboard and rack on each row. After the checkerboard comes the first *parity* dibit. Parity refers to a method of detecting (some) bitwise data errors, at the cost of using an additional bit (or, in this case, dibit). The first parity dibit is for detecting errors in the following odd-numbered data dibits; a second parity dibit for even-numbered dibits appears after the data dibits in the row, just prior to one or two white squares and the rack.

### Softstrip header

The first part of each Softstrip is the horizontal synchronization section. It includes the number of four-bit groups (nibbles) per row and is used to align the optical reader for scanning the strip. (A physical reader also uses this section to determine the contrast between paper and ink color, but that is not required for our work.) The horizontal synchronization section is followed by the vertical synchronization section, where the height of the dibits is encoded and is repeated multiple times per row. The vertical synchronization concludes with three zero bytes that indicate the start of the data.

### Data section

A file header with metadata is encoded first; it contains information about the encoded file. There are provisions for multiple files to be encoded in one strip, but we did not find any instances of that occurring. Full details about the file header can be found elsewhere (Cauzin Systems, Inc 1986b), but suffice it to say that the file header contains fairly typical file metadata: file name, length, type. Crucially for our purposes, the file header also contains a strip checksum, which is a strong(er) means of detecting data errors than parity alone.

## Corpus

The Softstrips we used were from five different sources and provided a wide spectrum of encoding densities and image quality.

### Digital generated Softstrips (Corpus1)

**Fig. 5:**
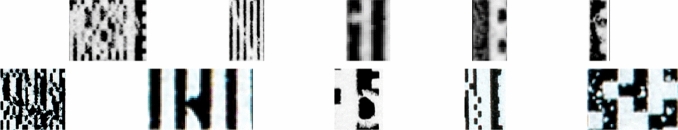
Corpus2 artifacts (above) and Corpus5 artifacts (below)

A Softstrip creation tool (Osborn 2016b) was used to generate Softstrips. Here the selection of input data could be arbitrary; we chose to use an icon collection, where each approximately $25\times 25$ pixel icon was encoded into a single Softstrip with a typical image resolution of $390 \times 2500$ pixels. This large dataset provided a baseline with optimal conditions to decode. Unfortunately, all of the following non-artificial datasets exhibit real-world problems: damaged dibits, missing dibit parts, white noise on black areas, damaged racks, and smeared printer ink. Figure 5 shows visual artifact samples from the *best*, professionally printed data sources below.

### Animated algorithms (Corpus2)

The book Animated Algorithms (1986) is one of the few books which used Softstrips. They were scanned at 300 DPI and have an image resolution of about $360\times 4200$ pixels.

### Cauzin Softstrip application notes and marketing material (Corpus3)

This collection was found on the Internet Archive (Cauzin Systems, Inc 1986c) with no DPI information; we extracted images from the PDFs. Many Softstrips in this collection contain some blurred areas.

### Magazine collection (Corpus4)

This collection consists of scans found online (Apple II Scans (n.d.) 2019) from various computer magazines and was processed similarly to Corpus3. Corpus4 contains by far the worst quality Softstrips, including some that would be difficult for humans to decode.

### StripWare (Corpus5)

Computer programs in the form of Cauzin Softstrips were sold in computer stores as booklets called StripWare. Corpus5 is comprised of a number of StripWare artifacts from eight booklets in very good condition (some still sealed in plastic). These Softstrips were scanned at 1200 DPI, with resolution ranging from about $750\times 6000$ pixels up to $800\times 9600$ pixels.

## Method and results

Overall, we tried four different methods of decoding Softstrips, beginning with ones that did not involve deep learning; full details are in Reimsbach (2018). The method described here is the second-best one, but this method used a simpler convolutional neural network (CNN) that is less prone to overfitting, and only decoded three fewer strips than a more complex CNN.

### Method

A single strip is selected using the Gimp image processing software and run through a three-step process: header processing, row extraction, and row decoding. Their description is followed by a discussion of the methods we implemented to improve the decoding of damaged strips.

#### Header processing

**Fig. 6:**
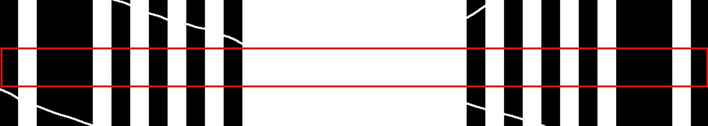
Damaged horizontal synchronization section

The horizontal synchronization section is the first part of a Softstrip and, in order to decode it, the white-to-black transitions need to be counted. Because a damaged horizontal synchronization section could have more white-to-black transitions than actually exist (Fig. 6), we first apply image noise reduction.

There is no guarantee that the noise reduction removes *all* the noise, however. Therefore, we use a heuristic distance measure to look for a group of self-similar pixel lines, such as the ones highlighted by the red rectangle in Fig. 6, and consider that a noise-free area. After an area with no noise is extracted, the decoder is then able to compute the number of nibbles per strip row.

A strip’s structure changes drastically from the horizontal to the vertical synchronization section and is reflected in the above features. Therefore, the distance measure is used again to locate the boundary between those sections.

#### Row extraction

A Softstrip image is divided into multiple segments, each 70 pixel lines, which are processed separately. The segmentation is to compensate for distortions in the scanned image.

To process each segment, the boundaries of the black checkerboard and the last rack square are first determined for each pixel line in the segment. After all pixel lines are scanned, the collected locations are filtered: every checkerboard location must be within the *checkerboard window*, which we make slightly larger than the actual checkerboard to help compensate for cases where the checkerboard is slightly shifted. In addition, every checkerboard and rack location must occur at least 6 times in the segment, in order to filter out noisy pixel lines. Once the boundaries are known, the checkerboard and rack pattern can be determined for each pixel line, using the color of the center pixel of the left checkerboard and last rack square.

After all segments are processed, adjacent pixel lines with the same valid pattern are grouped together into rows. There is no minimum number of pixel lines required to form a row, because in some cases only a single pixel line can be found in a row.

#### Row decoding

We used the Keras Python library along with a Tensorflow back end^1^ to develop a CNN-based row decoder. It was trained on data from five Corpus2 Softstrips whose rows we had decoded algorithmically (Reimsbach 2018): 17,094 1-dibit samples and 26,596 0-dibit samples, where 75% of the samples were used for training and 25% for testing. Almost 100% accuracy was reached after one forward/backward training pass (epoch).

Input to the CNN is a $20\times 20$ pixel grayscale image of a dibit. Recall that the number of nibbles per row is known from the strip’s header, and the row image to decode is divided by this value to get the average dibit size. If necessary, the row’s dibits are resized to make each one $20\times 20$ pixels. The CNN itself has six layers.

#### Handling damaged strips

We automatically apply three methods to attempt decoding of otherwise undecodable rows where parity checks have failed. As these are local improvements to make a row parity check succeed, all successful combinations must be stored and considered later during the checksum test.

*Row splitting* repeatedly partitions the row image horizontally in an effort to handle dibits whose damage falls in either the upper or lower areas of a partition. In the worst case, only a single pixel line in the entire row will lead to a correct result. *Row shifting* addresses the case where the start bar is damaged and the row image was incorrectly extracted as a result. Here, we try shifting the row 1–2 pixels to the left or right. Finally, *low confidence flipping* uses the confidence value output by the row-decoding CNN and tries flipping the dibits that were classified with low confidence ($< 90\%$).

### Results

Our method is able to successfully decode slightly over 91% of the corpora.^2^ This rises to over 99% if we exclude Corpus3 and Corpus4, and only consider images produced under controlled conditions. Obviously the generated strips of Corpus1 are a dominant factor, and if we exclude those the successful extraction drops to around 71%. However, this climbs again to almost 94% if we consider non-generated strips whose scanning we controlled. Overall, our experience with this method on different corpora is that the higher resolution strips gave a clear advantage when decoding, and even minor rotations of $0.01^\circ$ during strip selection could lead to decoding failures.

We performed a baseline validation of our decoder using Corpus1. Since the exact inputs were known, we decoded all the strips in Corpus1 and compared the results to the original input files; all matched exactly. Beyond that, we have defined successful decoding of a strip using the objective metric of having all of a strip’s parity bits and its checksum match, the two Softstrip mechanisms designed for this purpose. These mechanisms allegedly gave ‘an undetected bit error rate of less than one bit error per 10,000,000,000 bits’ (Cauzin Systems, Inc (n.d.a)).

Marketing claims notwithstanding, these are not strong tests in modern terms, and it is indeed possible for a strip to contain a combination of errors that cause the parity checks and checksum to succeed, or for there to be different readings that succeed under these criteria. We observe, though, that even in the case of errors it may be easy to reconstruct the intended data. For example, one Softstrip contained a text file whose checksum failed, producing the corrupted line ‘There is no$warranty.’ The correction here is obvious, and we argue that similar repairs of localized errors would be possible even with BASIC programs or binary code given semantic knowledge that is out of the scope of strip decoding. But using semantic knowledge is no different a situation than might be faced when interpreting writing on damaged, traditional physical artifacts; the important point is that our reader is able to extract strip content so that it may be seen and interpreted.

While it would be possible to attempt automated correction of failed data reads using, for instance, a trained text or code corpus, we would argue that it is best left as a separate process to be applied by the user of the data. While some types of Softstrip data are relatively robust, such as English text, computer code is not. A minor mistaken correction in code might easily yield nonfunctional code, or worse, code that apparently does function but produces incorrect results. Data correction is a task we feel is best left for a domain expert; the important part is to identify when such intervention may be necessary.

## Related work

One of the earliest bar codes for encoding software was the Paperbyte in 1977 (Budnick 1977), a 1-D barcode that was used to encode programs in Paperbyte books and Byte magazines. In 1983, the OSCAR 1-D bar code was released for encoding software in the Databar magazine, but only a single issue of it was ever published (Savetz et al. 2017). A modern OSCAR barcode reader is available (Teuwen 2016). However, this is a more straightforward decoding problem: OSCAR was a 1-D format, and the number of published OSCAR bar codes is very limited. We needed to handle many more strips and their visual artifacts.

Chris Osborn, the author of the Softstrip generator we used for Corpus1, used it to pose a challenge (Osborn 2016a): decode a generated Softstrip that contained the private key to a Bitcoin wallet. The winner did write a Softstrip reader (Sowerbutts 2016), but one that required a number of inputs from the user, only worked for strips under ideal conditions—and only needed to be able to read one particular Softstrip.

More broadly, there is prior work in cultural heritage on reading old media formats (Chenot et al. 2018) and work using CNNs (e.g., Can et al. 2018). Our work here adds to that body of knowledge. Also on point is preservation-focused archival work dealing, for example, with handling content on floppy disks (Durno and Trofimchuk 2015; Levi 2011), or extracting data from magnetic tapes (van der Knijff 2019). Many of the concerns echo our experience, with hardware and software either well past its best-before data, or missing entirely. We also note McNally’s advocacy for do-it-yourself solutions, albeit with a focus on born-digital content (McNally 2018); we feel our work is one answer to that call, for a medium that straddles both the physical and digital.

Parallels between our work and optical character recognition (OCR) can definitely be drawn. Indeed, a recent OCR survey observed that OCR work of late has been trending toward deep learning (Memon et al. 2020). The input for OCR is intended(!) to be legible to humans, of course, whereas the dibits of finer-resolution Softstrips are not easily discernable to the naked eye. Having said that, OCR must deal with an enormous variety of fonts and letter shapes well beyond the relatively fixed format of Softstrips; our point is that using deep learning to address this and similar tasks is now within the reach of non-specialists. There are certainly specific aspects of OCR, such as noise reduction, that are applicable in contexts like ours.

## Discussion and conclusion

What we have demonstrated with our digital reader for Cauzin Softstrips is that it is tractable to develop bespoke software solutions for obsolete media formats. We stress that neither of the authors are experts in neural networks, and yet we have seen excellent results simply using freely available Python libraries in a straightforward way. And, while Softstrips are arguably a niche, esoteric data format, in the bigger picture they and our digital reader act as a vehicle for more generally applicable lessons.

One important idea is how we decomposed the problem, abstracting away unnecessary functionality as well as breaking down the task until we were left with a well-defined classification problem. In our case, multiple Softstrips often appear on the same page, and instead of trying to automatically identify and process them all, we left the job of identifying single Softstrips to decode for a human. Similarly, we had initially considered having our software locate Softstrips’ positioning based on the alignment marks that the real physical reader would use, but eschewing that notion in favor of a human selecting the image region to process also simplified the software required. In other words, there is a “sweet spot” in design where, depending on the anticipated scale, it is quicker and easier to *not* automate things.

We can see the decomposition playing out further in our eventual decoding method, where parts of the Softstrip were tackled incrementally until we were left with the comparatively constrained task of classifying/reading dibits, albeit one made challenging by real-world conditions. Intuitively, one might reasonably think that our solution using deep learning is overkill, and that simpler algorithmic approaches would suffice. That is, in fact, what we initially thought—that was our starting point for this work, and an earlier independent attempt overseen by the second author not reported here. Ultimately those methods worked for a small number of Softstrips, but quickly broke down when faced with the range of damage present in some physical Softstrips along with the variant quality of Softstrip images not created under controlled conditions. CNNs, which learn relevant classification features by themselves and are known for image-based applications, presented an appealing, and surprisingly good, solution.

We do want to stress the helpfulness of having Cauzin information available in patents and other sources. Our task would not have been *im*possible without that information, but it would have required far more heroic efforts that would be beyond the reach of most non-specialists. Even so, there were some incompletely documented aspects of Softstrips that we needed to figure out on our own, but fortunately they were relatively minor ones.

The Cauzin Softstrip itself suffered a chicken-and-egg problem (Savetz et al. 2016). The limited number of available Softstrips did not attract enough customers to buy a Softstrip reader; magazines stopped publishing Softstrips because the target market was not big enough. Situations like these leave portions of our digital past locked into obsolete media formats, yet we restored access to the encoded data without need of a rare, functioning physical reader. While our decoding method was necessarily specific to the Softstrip, knowing the approach we used to read the strips, handle a wide variety of artifacts, and the lessons we learned are useful more generally.

Although analysis of the Softstrip corpora contents is outside the scope of this paper, we observe that this work speaks directly to archival accessibility. For the Cauzin Softstrip, despite them being physical artifacts, physical preservation is insufficient; it is only through being able to extract their data that the corpora become accessible for research. The Softstrips’ data can now be analyzed *en masse* to add to the growing body of scholarship on the history of personal computing (e.g., Halvorson 2020; Nooney et al. 2020; Rankin 2018), and close readings can be performed of individual Softstrips. For example, we came across a purported encryption utility program in the Softstrip collection, and by extracting its data using our software, we were able to study the program and determine its encryption method.

As an epilogue, we recently acquired a set of Softstrips from eBay, and with no adjustments whatsoever to our decoding software, we were able to decode nearly 95% of the 177 strips. This highlights both the robustness of our solution along with the utility of deep learning for archival and digital curation tasks.

## Footnotes

1 https://keras.io and https://www.tensorflow.org, respectively.

2 Corpus1 869/869; Corpus2 38/40; Corpus3 36/58; Corpus4 86/159; Corpus5 96/103; in total 1125/1229.
